# Doxorubicin-Induced Cardiac Toxicity Is Mediated by Lowering of Peroxisome Proliferator-Activated Receptor **δ** Expression in Rats

**DOI:** 10.1155/2013/456042

**Published:** 2013-02-28

**Authors:** Zhih-Cherng Chen, Li-Jen Chen, Juei-Tang Cheng

**Affiliations:** ^1^Department of Cardiology, Chi-Mei Medical Center, Yong Kang City, Tainan County 73101, Taiwan; ^2^Department of Medical Research, Chi-Mei Medical Center, Yong Kang City, Tainan County 73101, Taiwan; ^3^Department of Pharmacy, Chia Nan University of Pharmacy & Science, Jean-Tae City, Tainan County 71701, Taiwan; ^4^Institute of Basic Medical Sciences, College of Medicine, National Cheng Kung University, Tainan City 70101, Taiwan; ^5^Institute of Medical Sciences, Chang Jung Christian University, Quei-Ren, Tainan City 71101, Taiwan

## Abstract

The present study investigates the changes of peroxisome proliferator-activated receptors **δ** (PPAR**δ**) expression and troponin phosphorylation in heart of rats which were treated with doxorubicin (DOX). Wistar rats which were treated with DOX according to a previous method. The protein levels of PPAR**δ** and troponin phosphorylation were measured using Western blot. The PPAR**δ** expression in heart was markedly reduced in DOX-treated rats showing a marked decrease in cardiac dP/dT and cardiac output. Also, cardiac troponin phosphorylation was lowered in DOX-treated rats. Meanwhile, combined treatment with the agonist of PPAR**δ** (GW0742) reversed the decrease of cardiac dP/dT and cardiac output in DOX-treated rats. Then, primary cultured cardiomyocytes from neonatal rats were used to measure the changes of calcium concentration in cells. In addition to both decrease of PPAR**δ** expression and troponin phosphorylation in neonatal cardiomyocytes by DOX, a marked decrease of calcium concentration was also observed. Our results suggest the mediation of cardiac PPAR**δ** in DOX-induced cardiotoxicity in rats. Thus, activation of PPAR**δ** may restore the expression of p-TnI and the cardiac performance in DOX-induced cardio toxicity in rats.

## 1. Introduction

Doxorubicin (DOX) is a widely used chemotherapeutic agent in the treatment of tumors with a major side effect of cardiac toxicity [1, 2], which has not been effectively prevented by cardioprotective drugs [3, 4]. Thus, its clinical use is limited due to a cumulative dose-dependent cardiotoxicity including the electrocardiographic changes, arrhythmias, irreversible degenerative cardiomyopathy, and congestive heart failure [5–8]. The recent report showed that approximately 10% of patients treated with DOX or its derivatives will develop cardiac complications up to 10 years after cessation of chemotherapy [9]. Actually, the mechanism for cardiac toxicity caused by DOX or its metabolites is still not clear. Hypotheses regarding the cardiac toxicity of DOX include perturbation of calcium homeostasis, formation of iron complexes, and generation of radical oxygen species, mitochondrial dysfunction, and damage to cell membranes [10, 11].

PPARs are ligand-activated transcriptional factors that regulate expression of genes involved in lipid metabolism and inflammation [12]. Three subtypes of PPARs, PPAR*α*, PPAR*γ*, and PPAR*δ*, modulate the expressions of many genes and exert various bioactivities [12]. PPAR*α* is relatively abundant in tissues with a high oxidative capacity, such as liver and heart. PPAR*γ* expression is confined to a limited number of tissues, primarily adipose tissue [12, 13]. The ubiquitously expressed PPAR*δ* enhances the lipid catabolism in adipose tissue and muscle [12]. PPAR*δ*-dependent maintenance of inotropic function and metabolic effects is crucial for cardiomyocytes [14–16]. Deletion of cardiac PPAR*δ*, which is accompanied by decreased contraction, increased left ventricular end-diastolic pressure, and lowered cardiac output, leads to decreased contraction and increased incidence of cardiac failure [14].

Our previous study showed that cardiomyopathy in type-1 like diabetic rats is associated with a marked decrease in cardiac PPAR*δ* expression [17]. It seems possible that cardiac PPAR*δ* expression is involved in the cardiac toxicity of DOX. Thus, in the present study, we used Wistar rats and primary neonatal rat cardiomyocytes to investigate the role of PPAR*δ* in DOX-induced heart failure both *in vivo* and *in vitro*.

## 2. Materials and Methods

### 2.1. Materials

Doxorubicin from Sigma-Aldrich (St Louis, MO, USA) and GW0742 from Santa Cruz Biotechnology (Santa Cruz, CA, USA) were used. The fluorescent probe, Fura-2, was obtained from Molecular Probes (Eugene, OR, USA). Antibodies to PPAR*δ* and actin were purchased from Abcam (Cambridge, MA, USA). Antibodies to cardiac TnI and phospho-TnI (Ser 23/24) were purchased from Cell Signaling Technology (Beverly, MA, USA). 

### 2.2. Animal Model

All animal procedures were performed according to the Guide for the Care and Use of Laboratory Animals published by the US National Institutes of Health (NIH Publication No. 85-23, revised 1996), as well as the guidelines of the Animal Welfare Act. The male Wistar rats, weighing from 200 to 250 g, were obtained from the Animal Center of National Cheng Kung University Medical College. Heart failure was induced by intraperitoneal injection of 15 mg/kg DOX according to previous report [8]. It is well established that bolus injection of DOX (15 mg/kg) is enough to cause acute cardiomyopathy in rodent [18]. Otherwise, GW0742, a PPAR*δ*-specific activator [19], was dissolved in Dulbecco's modified Eagle's medium/dimethyl sulphoxide (DMSO) 6% (Gibco) and injected subcutaneously once a day at 1 mg/kg in DOX-treated rats. Another group of DOX-treated rats receiving same treatment with vehicle at same volume was used for comparison. Meanwhile, the age-matched normal rats receiving same treatments were taken as control. Then, under anesthesia with an inhalation of isoflurane (5%), they were cannulated in the right femoral artery with polyethylene catheters (PE-50). Mean arterial pressure (MAP) and heart rate (HR) were recorded using a polygraph (MP35, BIOPAC, Goleta, CA, USA). The rat's trachea was intubated for artificial ventilation (Small Animal Ventilator Model 683, Harvard Apparatus, Holliston, M) at 50 breaths/min with a tidal volume of 8 mL/kg and a positive end-expiratory pressure of 5 cm H_2_O. After incision into the rat's chest at the third intercostals space to expose the heart, a small section (1 cm long) of the ascending aorta was freed from the connective tissue. A Transonic Flow Probe (2.5PSB923, Transonic System Inc., Ithaca, NY, USA) was implanted around the root of the ascending aorta and connected to a Transonic transit-time blood flowmeter (T403, Transonic System Inc.). The cardiac output (CO) was calculated from the aortic blood flow, and the stroke volume (SV) was expressed as CO divided by HR. After the determination, the hearts were isolated from the anesthetized rats, rinsed with ice-cold phosphate-buffered saline (PBS), gently blotted, and weighed for western blotting analysis. All animal procedures were performed according to the Guide for the Care and Use of Laboratory Animals published by the US National Institutes of Health (NIH Publication No. 85-23, revised 1996), as well as the guidelines of the Animal Welfare Act.

### 2.3. Cell Culture and Treatment

Primary cultures of neonatal rat cardiomyocytes were prepared by a previous method [20] with modification. Briefly, under anesthesia with an inhalation of isoflurane (5%), the heart tissue from a 1 to 2 day-old Wistar rat was cut into 1- to 2-mm pieces and predigested with trypsin to remove the red blood cells. The heart tissue was then digested with 0.25% trypsin and 0.05% collagenase. The dissociated cells were placed in uncoated 10 cm dishes and incubated at 37°C in a 5% CO_2_ incubator for at least 1 h to remove the nonmyocyte cells. This procedure caused most of the fibroblasts to attach to the dishes, while most of the cardiomyocytes remained unattached. The population of cells enriched in cardiomyocytes was then collected and counted. The cells were cultured in DMEM (GIBCO BRL, Gaithersburg, MD, USA) with 1 mmol/L pyruvate, 10% fetal bovine serum (FBS), 100 units/mL penicillin, and 100 units/mL streptomycin. On the second day after plating, the medium was replaced. Three days after plating, the cells were exposed to hyperglycemic conditions as described in detail later. Animal handling and disposal were performed in accordance with NIH guidelines.

The DOX-treated cardiomyocytes were generated by treating the cells with 10^−9^~10^−6^ mol/L DOX for 24 h [21]. Then, cells were collected and subjected for Western blotting analysis. The treatment with GW0742 (PPAR*δ* agonist) was performed at 10^−6^ mol/L for 1 hour before the addition of DOX as described previously [15, 22].

### 2.4. Western Blotting Analysis

Protein was extracted from tissue homogenates and cell lysates using ice-cold RIPA buffer supplemented with phosphatase and protease inhibitors (50 mmol/L sodium vanadate, 0.5 mM phenylmethylsulphonyl fluoride, 2 mg/mL aprotinin, and 0.5 mg/mL leupeptin). Protein concentrations were determined with the Bio-Rad protein assay (Bio-Rad Laboratories, Inc., Hercules, CA, USA). Total proteins (30 *μ*g) were separated by SDS/polyacrylamide gel electrophoresis (10% acrylamide gel) using the Bio-Rad Mini-Protein II system. Protein was transferred to expanded polyvinylidene difluoride membranes (Pierce, Rockford, IL, USA) with a Bio-Rad Trans-Blot system. After transfer, the membranes were washed with PBS and blocked for 1 h at room temperature with 5% (w/v) skimmed milk powder in PBS. The manufacturer's instructions were followed for the primary antibody reactions. Blots were incubated overnight at 4°C with an immunoglobulin-G polyclonal rabbit anti-mouse antibody (Affinity BioReagents, Inc., Golden, CO, USA) (1 : 500) in 5% (w/v) skimmed milk powder dissolved in PBS/Tween 20 (0.5% by volume) to bind the PPAR*δ* in the heart specimens. The blot was incubated with goat polyclonal antibody (1 : 1000) to bind the actin serving as internal control. After the removal of primary antibody, the blots were extensively washed with PBS/Tween 20. The blots were then incubated for 2 h at room temperature with the appropriate peroxidase-conjugated secondary antibody diluted in 5% (w/v) of skimmed milk powder and dissolved in PBS/Tween 20. The blots were developed by autoradiography using the ECL-western blotting system (Amersham International, Buckinghamshire, UK). The immune blot of PPAR*δ* (49 kDa), actin (43 kDa), cardiac troponin (28 kDa), and phospho-troponin were quantified with a laser densitometer.

### 2.5. Measurement of Intracellular Calcium Concentration

The changes in intracellular calcium were detected using the fluorescent probe Fura-2 [23]. The neonatal cardiomyocytes were placed in buffered physiological saline solution containing 140 mM NaCl, 5.9 mM KCI, 1.2 mM CaCl_2_, 1.4 mM MgCl_2_, 11.5 mM glucose, 1.8 mM Na_2_HPO_4_, and 10 mM Hepes-Tris, to which was added 5 *μ*M fura-2, and incubated for 1 h in humidified 5% CO_2_ and 95% air at 37°C. The cells were washed and incubated an additional 30 min in PSS. The cells were inserted into a thermostated (37°C) cuvette containing 2 mL of calcium-free PSS. The fluorescence was continuously recorded using a fluorescence spectrofluorometer (Hitachi F-2000, Tokyo, Japan). Values of [Ca^2+^]*i* were calculated from the ratio *R* = *F*340/*F*380 by the formula: [Ca^2+^]*i* = Kd*B*(*R* − *R*_min⁡_)/(*R*_max⁡_ − *R*), where Kd is 225 nM, *F* is fluorescence, and *B* is the ratio of the fluorescence of the free dye to that of the Ca^2+^-bound dye measured at 380 nm. Background autofluorescence was measured in unloaded cells and subtracted from all measurements.

### 2.6. Catheterization for Hemodynamic dP/dt Measurement

Temporary pacing leads were used for short-term study and were placed in the right atrium and RV apex. A venogram imaged in 2 different angulations (left anterior oblique 30° and anteroposterior) was obtained to determine the anatomy of the coronary sinus venous system. An LV pacing electrode (IX-214; iWorx Systems, Inc., Dover, NH, USA) was placed either in the free wall region via the lateral or posterior vein or in the anterior region via the great cardiac vein. After femoral artery and venous puncture using the Seldinger technique [24], pressure transducer catheters were inserted into the heart to provide the RV, aortic, and LV pressures. Pressure catheters and pacing leads were connected to an external pacing computer (iWorx Systems, Inc., Dover, NH, USA) to execute the pacing protocol and to acquire hemodynamic signals.

### 2.7. Statistical Analysis

Data are expressed as the mean ± SEM for the number (*n*) of animals in one group as indicated. Statistical analysis was carried out using repeated measures analysis of variance (ANOVA) and Newman-Keuls post-hoc analysis. Bonferroni's correction was applied to the data, which were obtained from relatively small groups. A *P* value of 0.05 or less was considered significant.

## 3. Results

### 3.1. Effects of GW0742 on PPAR*δ* Expression and Cardiac Troponin I Phosphorylation in DOX-Treated Rats

**Figure 1:**
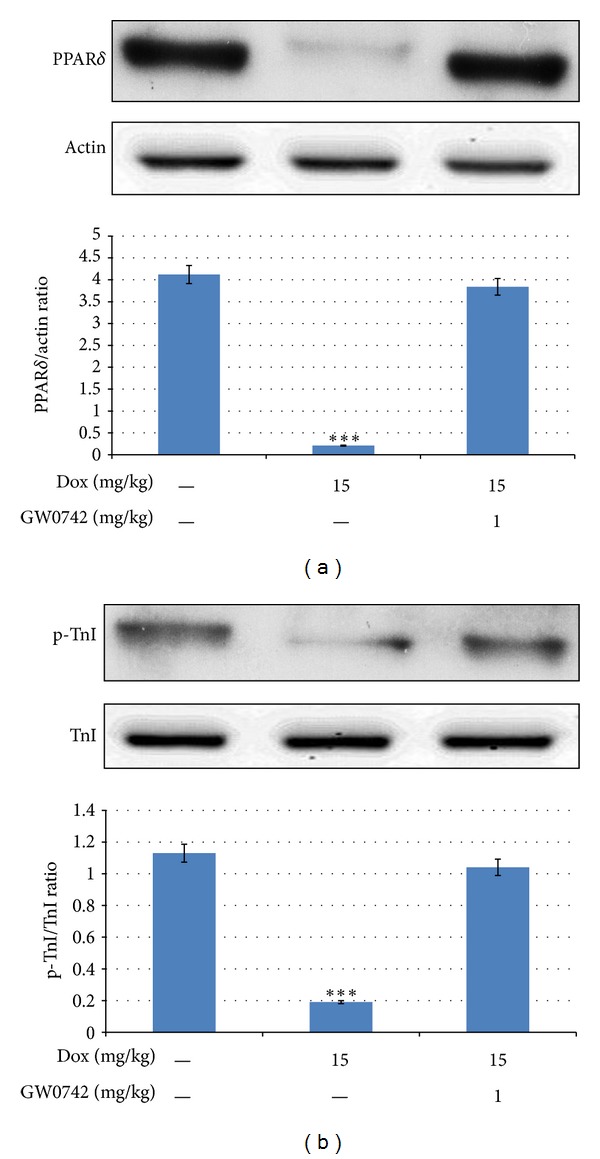
PPAR*δ* expression and TnI phosphorylation in hearts from DOX-treated rats, GW0742-added DOX-treated rats, or Wistar rats. Changes in cardiac PPAR*δ* protein expression (a) and the levels of cardiac TnI phosphorylation (b) were examined in age-matched Wistar rats (control rats), DOX-treated rats, and GW0742-added DOX-treated rats. All values are expressed as mean ± SEM (*n* = 6 per group). *P < 0.05 and **P < 0.01 as compared with Wistar rats.

The levels of PPAR*δ* protein expression and cardiac troponin I phosphorylation were significantly reduced in the heart of DOX-treated rats, compared with the control rats (Figure 1). Moreover, the decrease in expression of PPAR*δ* or cardiac Troponin I phosphorylation was markedly reversed by GW0742 in DOX-treated rats (Figure 1).

### 3.2. Improvement of Cardiac Function in DOX-Treated Rats by GW0742

**Figure 2:**
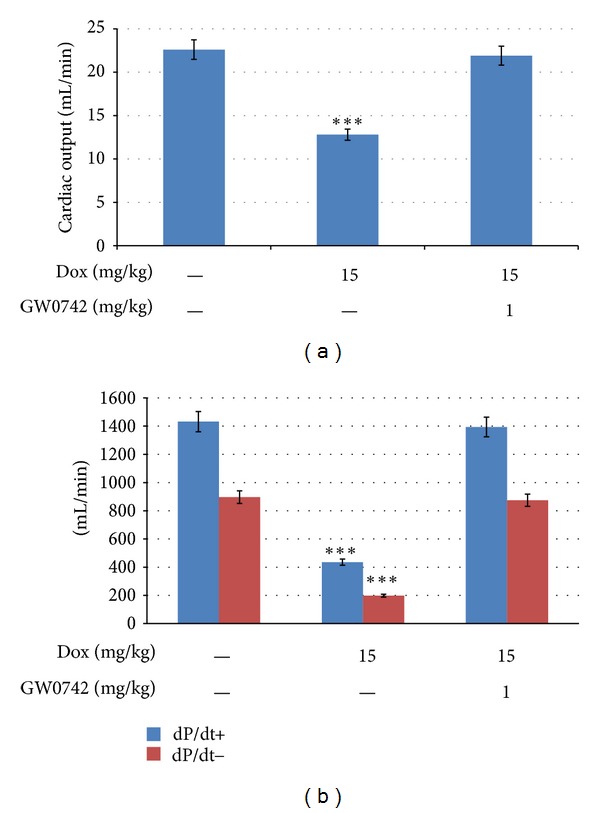
Effect of GW0742 on cardiac output and hemodynamic dP/dt in DOX-treated rats. Changes in cardiac output (a) and hemodynamic dP/dt (b) in DOX-treated rats by a 3-day treatment of GW0742 (1 mg/kg, S.C). Data represent mean ± SE of six experiments. **P < 0.01 as compared with control group.

The values of cardiac output in addition to maxdP/dt and mindP/dt were significantly (*P* < 0.001) reduced in DOX-treated rats as compared with the control rats. Also, the decrease in cardiac output or maxdP/dt and mindP/dt was markedly reversed in DOX-treated rats after a 3-day treatment with GW0742 (1 mg/kg), as shown in Figures 2(a) and 2(b). 

### 3.3. Effects of Doxorubicin on the Reduction of PPAR*δ* Expression and Cardiac Troponin I Phosphorylation in Neonatal Rat Cardiomyocytes

**Figure 3:**
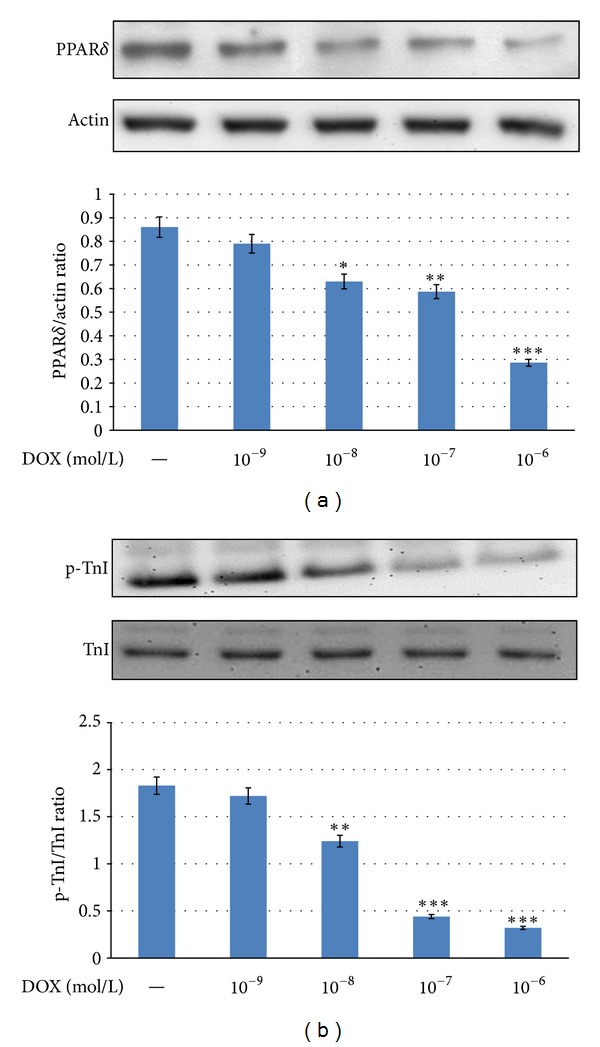
Effects of doxorubicin on PPAR*δ* expression and cardiac troponin I phosphorylation in neonatal rat cardiomyocytes. Cardiomyocytes from neonatal rats were cultured with 10^−9^~10^−6^ mol/L doxorubicin for 24 h. These cells were harvested to determine the expression of PPAR*δ* (a) and the level of troponin I phosphorylation (b) using Western blotting analysis. All values are presented as mean ± SEM (*n* = 6 per group). *P < 0.05, **P < 0.01, and ***P < 0.001 as compared with control cells.

We used primary neonatal rat cardiomyocytes to investigate the effects of DOX on expression of PPAR*δ* and cardiac Troponin I phosphorylation. After the incubation with 10^−9^~10^−6^ mol/L DOX for 24 h, the cells were harvested to compare the expression of PPAR*δ* and the level of cardiac Troponin I phosphorylation in cells. The expression of PPAR*δ* protein and the level of cardiac troponin I phosphorylation in neonatal rat cardiomyocytes were significantly reduced by DOX treatment in a concentration-related manner (Figure 3). Then, the most effective dose (10^−6^ mol/L) of DOX was used in the fallowing studies.

### 3.4. Effects of GW0742 on PPAR*δ* Expression and Cardiac Troponin I Phosphorylation in DOX-Treated Neonatal Rat Cardiomyocytes

**Figure 4:**
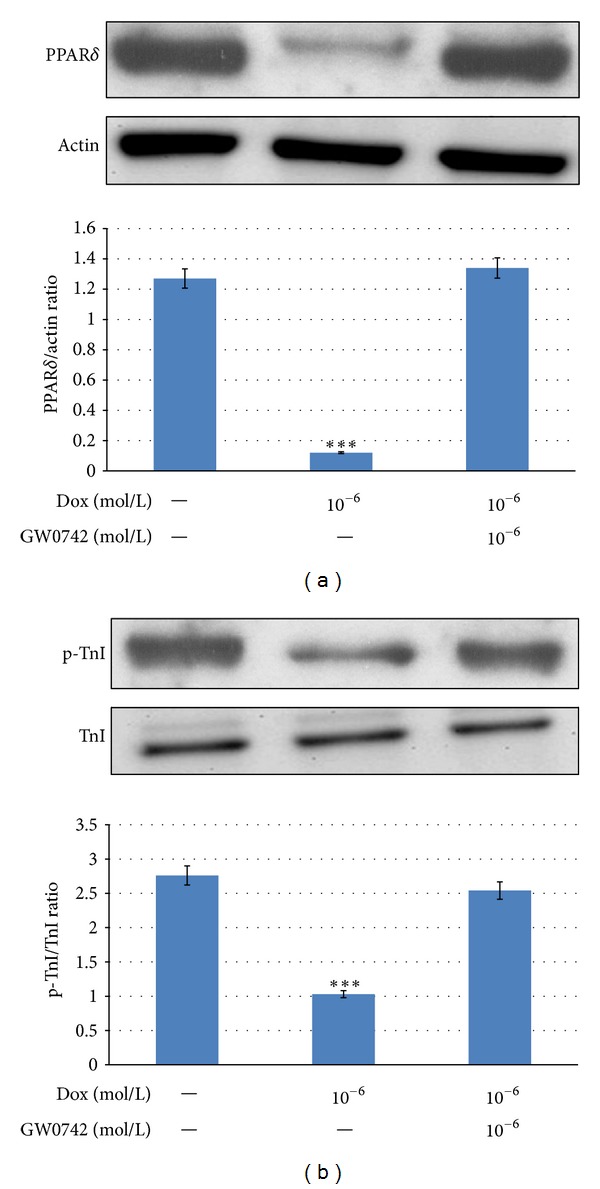
Effects of GW0742 on PPAR*δ* expression and cardiac troponin I phosphorylation in doxorubicin-treated neonatal rat cardiomyocytes. Changes of PPAR*δ* expression (a) and cardiac troponin I phosphorylation (b) in DOX-treated cells by GW0742. Cells were treated with 10^−6^ mol/L GW0742 for 1 hour before the incubation with 10^−6^ mol/L DOX. After 24 h, they were harvested for measuring PPAR*δ* protein expression (a) and cardiac troponin I phosphorylation (b) by Western blot. All values are expressed as mean ± SEM (*n* = 6 per group). *P < 0.05, **P < 0.01, and ***P < 0.001 as compared with DOX-treated cells.

The decrease in expression of PPAR*δ* protein or cardiac Troponin I phosphorylation was restored by treatment with GW0742 in DOX-treated cells, respectively (Figure 4).

### 3.5. Effects of GW0742 on the Intracellular Concentration of Calcium in DOX-Treated Neonatal Rat Cardiomyocytes

**Figure 5:**
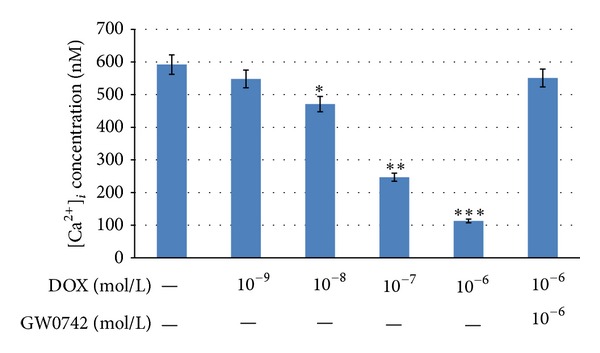
Effects of GW0742 on intracellular calcium release in DOX-treated neonatal rat cardiomyocytes. Changes in intracellular calcium concentration were detected by Fura-2 using a fluorescence spectrofluorometer. Cells were treated with 10^−6^ mol/L GW0742 for 1 hour before incubation with 10^−6^ mol/L DOX and then used for measuring intracellular calcium concentration. All values are expressed as mean ± SEM (*n* = 6 per group). *P < 0.05, **P < 0.01, and ***P < 0.001 as compared with DOX-treated cells.

The fluorescent probe, fura2-AM, was used to detect the intracellular calcium concentration in DOX-treated neonatal rat cardiomyocytes. As compared with control, GW0742 restored the intracellular calcium concentration in DOX-treated cardiomyocytes (Figure 5).

## 4. Discussion

The present study found that gene expression of PPAR*δ* is reduced in rats with doxorubicin-(DOX-) induced cardiac toxicity. Also, activation of PPAR*δ* may improve the cardiac performance damaged by DOX. Thus, we suggest the first report regarding the important role of PPAR*δ* in DOX-induced cardiac toxicity.

According to a previous method [25], we established animal model showing DOX-induced cardiac toxicity that has been confirmed using the marked decrease in cardiac output and contraction (dP/dt) in addition to the lowering of cardiac Troponin I phosphorylation. Also, gene expression of cardiac PPAR*δ* is markedly reduced in this animal model. Mediation of PPAR*δ* in the lowering of cardiac performance induced by DOX can thus be considered. Actually, we found that a 3-day treatment with GW0742 at the dose sufficient to activate PPAR*δ* reverses the cardiac performance in DOX-treated rats. This is consistent with the view that cardiac PPAR*δ* is mediated in contraction of heart and decrease of PPAR*δ* is related to higher incidence of cardiac failure [14]. 

Then, we used the primary cultured cardiomyocytes from neonatal rats to investigate the potential mechanism(s). Although cardiac contraction was not determined, Troponin I phosphorylation was reduced in cardiomyocytes by DOX in a concentration-related manner. The reduced phosphorylation of Troponin I has been identified in the failing hearts of human studies [26]. Thus, the reduced Troponin I phosphorylation may indicate the contractile defects as shown in hearts induced by DOX. Moreover, PPAR*δ* expression is also parallel reduced by DOX in cardiomyocytes. DOX is one of the widely used agents for the treatment of cancer with a limitation in clinical utility due to the irreversible cardiac toxicity [2, 27, 28]. In the current work, we demonstrated that DOX impaired cardiac function with a decrease in cardiac PPAR*δ* expression both *in vivo* and *in vitro*.

The pathological mechanism of DOX-related late cardiotoxicity is multifactorial [29], but the prevalent hypothesis ascribes the dominant role to oxidative stress linked to redox-cycling of the drug [30, 31]. The DOX redox-cycling is started from one-electron reduction with the formation of DOX radical (DOX*) [32]. Previous study has shown that greater amount of reactive oxygen species (ROS) could reduce the PPAR*δ* expression in the heart of rats under hyperglycemic condition [17]. In this work, we observed that PPAR*δ* expression is reduced by DOX in rats and cardiomyocytes and the redox unbalance induced by DOX may apply a possible mechanism of repression of PPAR*δ*.

Both cardiac output and cardiac contraction identified using maxdP/dt and mindP/dt decreased in DOX-treated rats. A decrease in cardiac performance induced by DOX is characterized as described in previous reports [33]. Then, in the *in vitro *study, intracellular calcium concentration was also reduced in cardiomyocytes by DOX in a dose-related manner. This is consistent with the previous report [34, 35]. Moreover, this change was reversed by GW0742 at concentration sufficient to activated PPAR*δ*. The new view regarding the important role of PPAR*δ* in DOX-induced cardiac toxicity can thus be considered.

Previous studies have demonstrated that Troponin I phosphorylation most likely acts through an enhanced off rate during Ca^2+^ exchange with Troponin C, leading to acceleration of relaxation and an increase in cardiac output [26, 36–39]. Recent studies also demonstrated that direct activation of PPAR*δ* by GW0742 may result in higher of TnI phosphorylation and cardiac performance. This action could be reversed after the treatment of calcium chelators [40–43]. A decrease in cardiac intracellular calcium may result in the lowering of Troponin I phosphorylation and this view has been identified in the present study. Furthermore, Troponin I phosphorylation was also reversed by GW0742 in the hearts of DOX-treated rats. Also, treat with GW0742 at effective dose as described previously [44, 45] was observed to improve cardiac performance in DOX-induced cardiotoxicity in rats. Taken together, it is suitable to speculate that the cardiotonic effect of GW0742 on DOX-induced cardiotoxicity seems related to an increase of Ca^2+^ concentration.

## 5. Conclusions

Cardiac PPAR*δ* plays an important role in DOX-induced cardiotoxicity in rats. Thus, activation of PPAR*δ* may restore the expression of p-TnI and the cardiac performance in DOX-induced cardiotoxicity in rats.
